# An enhanced approach to minimum variance unbiased velocity estimation, incorporating horizontal and vertical handoff in HetNets

**DOI:** 10.1038/s41598-025-16080-8

**Published:** 2025-08-26

**Authors:** Ravi Tiwari, Amit Kumar Rahul, Manoj Kumar Singh, Om Namha Shivay, S. Sivakumar, Saurabh Chandra Maury

**Affiliations:** 1 School of Electronics Engineering (SENSE), Vellore Institute of Technology (VIT), Chennai, Tamil Nadu India; 2 Department of Mathematics, School of Advanced Sciences (SAS), Vellore Institute of Technology (VIT), Chennai, Tamil Nadu India; 3 P.G. Department of Mathematics, Magadh University, Bodh Gaya, Bihar India

**Keywords:** Base station density, Cramer-Rao lower bound, Heterogeneous network, Mobility management, Minimum variance unbiased estimation, Probability mass function, User velocity, Electrical and electronic engineering, Engineering, Mathematics and computing

## Abstract

This paper presents an enhanced approach to Minimum Variance Unbiased (MVU) velocity estimation in Heterogeneous-Networks (HetNets) by addressing horizontal and vertical handoffs. In HetNets, the abundance of base stations (BSs) results in frequent unnecessary handoffs and service disruptions for mobile users, posing challenges for mobility management. Accurate velocity estimation is crucial for effective mobility management. Our proposed strategy involves tracking vertical and horizontal handoffs over a specified time interval. Through mathematical modeling, we approximate the analytical expression of the handover count probability-mass-function in HetNets as Rayleigh distributed and calculate its scale parameter based on velocity, BS density, and measurement time span. We derive the Cramer-Rao lower bound (CRLB) and utilize the Neyman-Fisher factorization method to obtain the sufficient statistics. Leveraging the Rao-Blackwell-Lehmann-Scheffe (RBLS) theorem, we derive the MVU estimator. Our results demonstrate a close alignment between the proposed estimator’s variance and the CRLB. Furthermore, we observe that increased user velocity leads to higher velocity estimation variance, indicating greater challenges in accurate estimation for faster-moving users. Simulation results show that a higher BS density and longer handover measurement periods can substantially reduce velocity estimation errors, highlighting the benefits of an improved HetNet infrastructure and extended measurement durations for precise velocity estimation.

## Introduction

In the last few decades, wireless communication systems have undergone remarkable advancements^1^. The proliferation of data-intensive applications in modern mobile devices has resulted in an exponential surge in cellular data traffic^2^. Technological innovations have also led to a significant increase in mobile data traffic generated by the latest wireless devices^3^. To meet the escalating demand for high data rates and seamless mobility, future wireless networks must be designed to accommodate large numbers of users. These networks should be capable of efficiently handling the increasing data traffic while ensuring uninterrupted connectivity and fast data transfer speeds^4^.

Researchers are actively developing technologies to overcome future challenges in cellular network deployment. These technologies can be categorized into several areas:

- Spatial Reuse through Network Densification: This involves maximizing spatial reuse by deploying small cell BSs alongside existing macrocellular network infrastructure. By increasing the number of base stations, network coverage and capacity can be significantly improved^5^.
- Exploration of Higher Usable Bandwidths: Researchers are exploring the use of higher carrier frequencies within both licensed and unlicensed spectrum. By utilizing these higher frequencies, networks can benefit from larger bandwidths, resulting in increased data rates and improved network capacity^6^.
- Spectral Efficiency Improvement Techniques: Several techniques are being researched to enhance spectral efficiency like Orthogonal Frequency Division Multiplexing (OFDM)^7^, Cooperative Communication, Dynamic Time Division Duplexing (TDD)^8^, Massive Multiple Input Multiple Output (MIMO)^9^; etc.

Network densification, specifically through spatial reuse, is emerging as a highly promising solution to address the escalating demands of rapidly increasing data traffic^10^. In order to achieve spatial reuse, network providers are strategically deploying small BSs in the existing macro-cell networks. This approach effectively increases the spectrum efficiency of the network. The deployment of small cell BSs alongside macro cell BSs creates a HetNet, where both types of BSs coexist harmoniously. This HetNet configuration enables a more efficient utilization of resources, resulting in enhanced network performance and capacity^11,12^.

Small cells are wireless networks that operate in both licensed and unlicensed spectrum and have shorter ranges compared to high-power macro cell BSs. These small cells are designed to provide wireless service both indoors and outdoors, particularly in areas with high data traffic^13^. Macro cell BSs, on the other hand, offer larger coverage areas^14^. In addition to varying transmission power levels, heterogeneous networks (HetNets) also feature different radio access technologies. These can include Bluetooth, Wireless Fidelity (Wi-Fi), 3G, Long term evaluation (LTE), 4G, 5G, and 6G. The deployment of macro BSs involves careful planning that takes into consideration geographic locations and coverage requirements. On the contrary, small cell BSs are deployed within macrocellular networks to address coverage gaps and areas with high traffic demands. As a result, small cell BSs are often deployed randomly, leading to dense local frequency reuse and improved network capacity^15^.

HetNets have demonstrated significant potential in contributing to the ambitious goal of achieving a 1000-fold increase in the capacity of next-generation cellular networks^16^. However, as BSs become more densely distributed, mobile users often experience service disruptions, posing a critical challenge for effective mobility management in HetNets^17^. Handover represents the most effective mechanism for addressing mobility issues by transferring ongoing calls from one BS to another without call drops. There are two primary types of handovers: horizontal and vertical. Horizontal handover pertains to the transfer of a mobile device’s connection within the same type of network. In contrast, vertical handover entails transitioning between different types of networks, such as transitioning from a cellular network to a Wi-Fi network, or from a Wi-Fi network to another type of network^18^.

The velocity of mobile users plays a vital role in the handover process, making it essential to gather this information for successful mobility management. Additionally, user velocity data can be leveraged for various purposes, including load balancing, scheduling, and energy management^19^. By utilizing user velocity data intelligently, network operators can optimize network performance and enhance the overall user experience in HetNet deployments.

With the advancements in mobile devices, various sensors such as Global Positioning System (GPS) and Wi-Fi have become available for velocity estimation^20^. However, these sensors may not be accessible in all areas. For instance, Wi-Fi signals are often unavailable in rural areas, and GPS signals can be weak in cities. Additionally, the limited power supply of mobile devices poses a challenge for relying solely on these sensor technologies for velocity estimation in HetNets. Another technique for velocity estimation is Doppler estimation^21^, which utilizes the Doppler effect. However, its complexity and standardization issues have hindered its widespread adoption in cellular network standards. For accurate velocity estimation in HetNets, it is crucial to have methods that can calculate the user’s velocity at the service provider’s location. This research paper aims to explore and evaluate such methods, providing insights and serving as a guide for future research in this area. By developing reliable velocity estimation techniques at the service provider’s end, HetNets can improve mobility management and enhance the overall network performance.

In the context of LTE Advanced, the 3rd Generation Partnership Project (3GPP) has been working on improving LTE performance through measures like carrier aggregation and HetNets^22^. The LTE specifications in Release-8^23^ allow service providers to utilize handover-count measurements for estimating and detecting mobility states. Several studies in the literature have explored the use of handover-count measurements for mobility state detection. In one such study^24^, the authors conducted a simulation-based analysis to evaluate mobility. They assigned different weights to handover counts for macro-to-small-cell and small-to-macro-cell base station handovers. This approach aimed to enhance the accuracy of user velocity estimation. By incorporating such weighted handover count analysis, service providers can improve the estimation of user velocity and further optimize mobility management in LTE Advanced systems. These advancements contribute to maximizing network performance and enhancing the overall user experience.

In the work presented in^25^, the authors propose a random waypoint mobility model for regular and stochastic geometry-based BS deployments. Based on this model, they derive the mean number of handovers. In another study,^26^, the authors derive closed-form expressions for the handover rate. Their analysis considers an independent Poisson-point-process (PPP) with arbitrary transmit power, path-loss exponent, and spatial density for each tier. This allows for a comprehensive understanding of handover dynamics in multilayer networks. Furthermore, in^27^, the authors derive a closed-form expression for the cross-tier handover rate in a two-tier network, utilizing stochastic geometry. This model specifically focuses on vertical handovers, which occur when users move between macro-cell and small cell base stations. The work presented in^28^ delves into the handover rate and coverage probability of users in HetNets, incorporating the consideration of handover failures. This analysis provides valuable insights into the performance of HetNets from a handover perspective. Moreover, studies such as^29,30^, and^31^ propose the use of handover-count samples for velocity estimation by service providers. These approaches aim to leverage the information available from handover counts to estimate user velocity accurately. These various studies contribute to enhancing our understanding of handover dynamics, velocity estimation techniques, and overall performance analysis in HetNets.

The authors in^29,30^ proposed and demonstrated the practicality of a handover-count based velocity estimation approach in HetNets. This technique approximated the probability mass function (PMF) of vertical handover-count samples using Gamma and Gaussian distribution functions. Subsequently,^32,33^ developed a velocity estimator based on Gamma distributed handover count measurements. It’s important to note that the maximum likelihood (ML) estimator is advantageous in situations where a MVU estimator either does not exist or is challenging to investigate. Additionally, the ML estimator is efficient only when a large number of measurements are available. The research considered both horizontal and vertical handoff for handover count PMF calculations to provide a more realistic representation. From the literature discussed, it can be inferred that there is still a need for further investigation into an MVU estimate for user velocity while utilizing handover-count measurements that take into account both horizontal and vertical handoff scenarios.

The primary research contribution of this study involves delving into an unexplored domain focused on deriving an MVU estimator for user velocity based on handover count PMF, taking into account both horizontal and vertical handoffs. The study considers the accumulation of horizontal and vertical handover counts over a specified time frame as an indication of velocity and approximates its PMF using the Rayleigh distribution. The emphasis on MVU estimation stems from its unbiased nature, ensuring that the mean of the estimate aligns with the actual value of the unknown parameter (user velocity) while exhibiting minimal variance compared to other unbiased estimators. Furthermore, the utilization of the Rayleigh distribution for approximating the PMF of handover-count samples is justified by its low Mean Square Error (MSE) when compared to simulated handover-count PMF incorporating both horizontal and vertical handovers. Notably, this area of inquiry appears to be unprecedented in existing literature. In summary, the key research contribution of this work can be articulated as follows:

- We develop the system model by drawing on the insights provided in the analyses presented in references^29–31^, while considering the availability of handover count measurements at the service provider end. In our model, we make the assumption that unplanned microcell BS locations conform to a PPP, and their corresponding coverage areas are represented by a Poisson Voronoi Tessellation^34^. Furthermore, our calculations for handover count PMF take into account both horizontal and vertical handoffs.
- We estimated the PMF of handover count by employing a Rayleigh distribution and determining the scale parameter of the distribution based on the Minimum Mean Square Error (MSE) between the estimated PMF and the simulated PMF.
- By employing the Neyman-Fisher factorization method, we derive the sufficient statistics required to estimate user velocity.
- Subsequently, utilizing the RBLS theorem, we introduce an MVU estimator for user velocity based on Rayleigh-distributed handover counts in HetNets.
- Ultimately, to evaluate the MVU estimate’s performance, we compute its estimator variance and the CRLB on the minimum error variance.

**Fig. 1:**
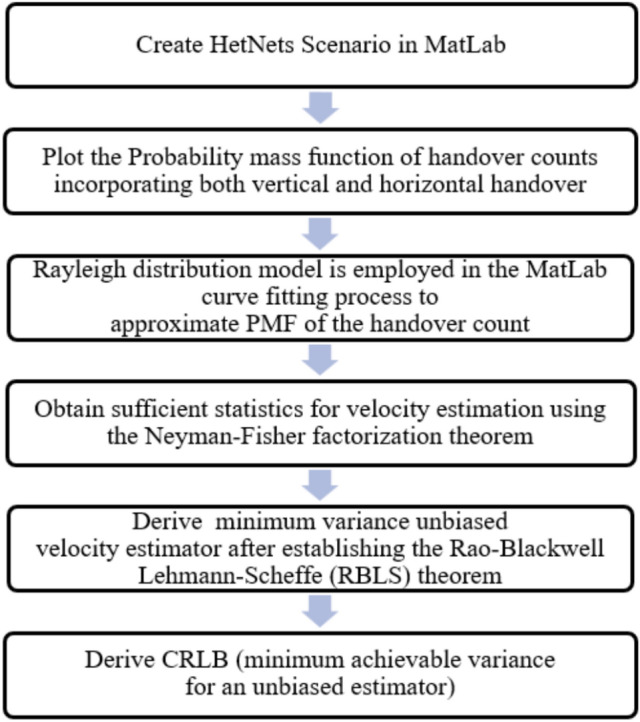
Workflow chart for velocity estimation in heterogeneous networks.

Figure 1 shows the workflow chart for estimating user velocity in HetNets. The process begins by creating a HetNets scenario in MATLAB, followed by plotting the PMF of handover counts that account for both vertical and horizontal handovers. The Rayleigh distribution model is then applied through MATLAB’s curve fitting tools to approximate the PMF of the handover count. Next, sufficient statistics for velocity estimation are obtained using the Neyman-Fisher factorization theorem. Building on this, the minimum variance unbiased velocity estimator is derived after establishing the RBLS theorem. Finally, the CRLB is derived to determine the minimum achievable variance for any unbiased estimator, providing a benchmark for the estimation method’s performance.

The enhanced MVU velocity estimation approach presented in this manuscript offers significant real-world applicability across modern wireless communication systems, especially within HetNets. Accurate user velocity information facilitates more efficient mobility management in cellular networks by enabling smarter handover decisions, which minimizes unnecessary handoffs and service interruptions: an essential factor for maintaining seamless connectivity in environments densely populated with both macro and small cells. This methodology also finds utility in urban mobility and intelligent transportation, where its integration into 5G-V2X (Vehicle-to-Everything) systems supports real-time vehicular safety applications, such as adaptive handover management for connected vehicles navigating complex urban landscapes. Furthermore, network planning and optimization can benefit from this framework, as service providers are empowered to optimize base station placement, adjust handover parameters, and improve load balancing strategies, all of which contribute to superior overall network performance and a better user experience.

The paper is organized as follows: Sect. 2 provides the system model for deploying small cell BSs within macro cells using stochastic geometry, including the approximation of the PMF of handover count considering both horizontal and vertical handoffs. In Sect. 3, the Neyman-Fisher factorization method is utilized to derive sufficient statistics for computing the estimate of user velocity. Section 4 covers the derivation of a MVU estimator based on the approximated PMF of handover count through the RBLS theorem. Additionally, Sect. 5 calculates the Cramer-Rao lower bound for the velocity estimator based on horizontal and vertical handover counts samples. The proposed velocity estimation approach is validated through numerical results in Sect. 6, followed by a conclusion in Sect. 7.

## System model

Due to the lack of an exact PMF of handover-count in literature and the challenges associated with the mathematical solution, a handover-based estimator requires an approximate PMF of the handover-count over a pre-defined time span. Here we are modeling the statistics on the number of handover-count in HetNet. The location of BS in HetNets is a 2D random process, so the measurements are also random, and therefore we use statistical modeling to analyze and evaluate performance in HetNets.

**Fig. 2:**
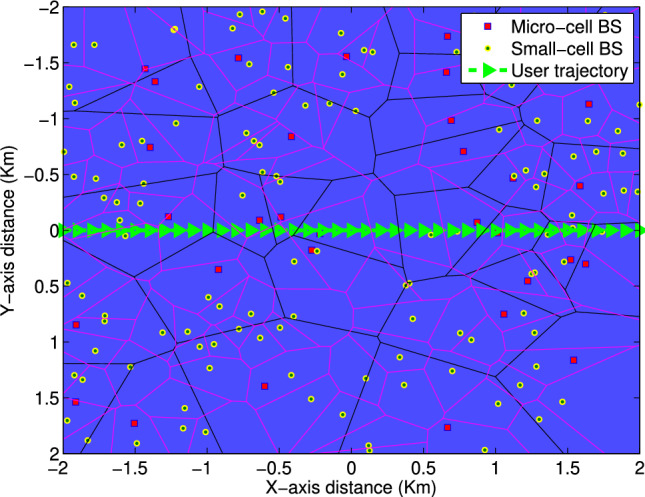
Stochastic Geometry of HetNets with user trajectory.

The research presented in this paper draws inspiration from the system model proposed in^29^ and^30^, which posits that macro-cell and small-cell base stations (BSs) operate on different frequency bands. The positioning of both types of BSs is depicted using a PPP, where the coverage area of small-cell BSs is independent of macro-cell BSs due to the utilization of distinct frequencies. In assessing the number of horizontal and vertical handoffs, the dedicated channel deployment of small-cell BSs, represented through Poisson-Voronoi tessellation^35^, has been considered within their respective coverage areas.

Figure 2 illustrates a scenario in HetNets where small-cell BSs are nested within the macro-cell network. The locations of the small-cell BSs are represented using a PPP with an intensity of $\lambda$ $BSs/km^2$, and their coverage is depicted by Poisson-Voronoi tessellation. User movements are assumed to follow straight trajectories, although alternative non-linear paths may be approximated as piecewise linear trajectories. The user’s boundary crossings are denoted as handovers, where the handover process involves the seamless transfer of ongoing services to another service provider. The handover counting process encompasses both small-cell to small-cell handovers (horizontal handovers) and small-cell to macro-cell handovers (vertical handovers). Additionally, small-cell BSs are prioritized in user allocation, resulting in most users being associated with the small-cell network.

**Fig. 3:**
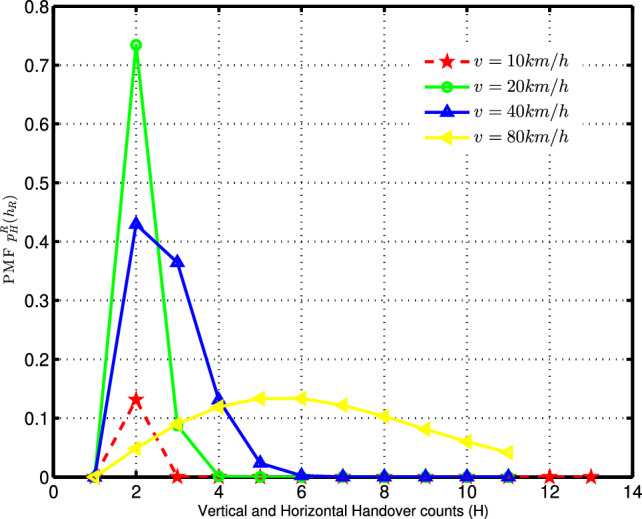
Probability mass function of handover counts for $\lambda =100\ \text{BSs/km}^2$ and $T=8s$.

This paper utilizes stochastic geometry to model the dense deployment of small cells and macro-cell BSs, illustrated in Fig. 2. The simulated PMF plot of handover counts $(p_H^R (h_R ))$, incorporating both horizontal and vertical handoffs, is presented in Fig. 3. In this scenario, the BS density is $\lambda = 100BSs/km^2$ and the measurement time-span is $T = 8s$. The random variable *H* is used to denote the handover counts.

### Utilizing the rayleigh distribution model for approximating handover-count PMF

The Rayleigh distribution model is employed in the Matlab curve fitting process to approximate PMF of the handover count as,

1 $$\begin{aligned} p_H^R\left( h_R\right) =\frac{\psi (h_R)}{\eta ^2}exp{\left( -\frac{\psi ^2\left( h_R\right) }{2\eta ^2}\right) },\ for\ \ h_R\in \left\{ 1,2,3,\ldots\right\} \end{aligned}$$

where $\psi (h_R)$ denotes a function of $h_R$ that represents the count of handovers within a specified time interval. In this context, the symbol *R* signifies the Rayleigh distribution, while $\eta$ denotes the scaling parameter associated with the Rayleigh distribution.

The accuracy of the approximated PMF can be evaluated by computing the MSE between the simulated and approximated PMF as,

2 $$\begin{aligned} MSE=\frac{1}{N_{h_R}}\sum _{h_{R=1}}^{N_{h_R}}\left( p_H^R\left( h_R\right) -p_{h_R}\left( h_R\right) \right) ^2 \end{aligned}$$

where $N_{h_R}$ is the number of points in PMF. The parameter $\eta$ is computed to minimize MSE between the simulated PMF and the Rayleigh approximated PMF, and it can be expressed as follows:

3 $$\begin{aligned} \eta ^2=\frac{1}{2}\left( 0.41\upsilon T\sqrt{\lambda }+0.07+\left( \frac{4\upsilon T\sqrt{\lambda }}{\pi }\right) ^2\right) \end{aligned}$$

where *T* represents the time period utilized for handover-count measurement; $\upsilon$ denotes the velocity of users; and $\lambda$ represents the density of BSs. In the following section, we obtain sufficient statistics for velocity by leveraging the Neyman-Fisher factorization theorem.

## Sufficient statistics for velocity estimation

In order to obtain sufficient statistics for velocity estimation, we analyze the approximated PMF of the handover-count samples outlined in Eq. (1). Let $\mathbf{h}_\mathbf{R}=\left[ {h_R}_j:j=1,2,3,\ldots,K-1\right]$ represent a vector of *K* independent and identically distributed (i.i.d.) handover count samples. The joint PMF of these *K* handover count samples can be expressed as follows,

4 $$\begin{aligned} p_H^R\left( \mathbf{h}_\mathbf{R}\right) =\prod _{j=0}^{K-1}{p_H^R\left( {h_R}_j\right) } \end{aligned}$$

Using Eq. (1), we substitute the value of $p_H^R\left( h_R\right)$ into Eq. (4), yielding

5 $$\begin{aligned} p_H^R\left( \mathbf{h}_\mathbf{R}\right) =\prod _{j=0}^{K-1}{\frac{\psi ({h_R}_j)}{\eta ^2}exp{\left( -\frac{\psi ^2\left( {h_R}_j\right) }{2\eta ^2}\right) }}. \end{aligned}$$

Upon further simplification of Eq. (5), we obtain

6 $$\begin{aligned} p_H^R\left( \mathbf{h}_\mathbf{R}\right) =\left( \frac{\psi ({h_R}_j)}{\eta ^2}\right) ^Kexp{\left( -\sum _{j=0}^{K-1}\frac{\psi ^2\left( {h_R}_j\right) }{2\eta ^2}\right) } \end{aligned}$$

Now, to extract sufficient statistics using the Neyman-Fisher factorization theorem, we aim to initially break down the PMF of handover-counts into two factors as,

7 $$\begin{aligned} p_H^R\left( \mathbf{h}_\mathbf{R}\right) =\left[ f\left( B\left( {h_R}_j\right) \right) ,\ \upsilon \right] \left[ G\left( {h_R}_j\right) \right] \end{aligned}$$

such that, *f*(.) is a function depending on ${h_R}_j$ only through $B({h_R}_j)$ and *G*(.) is a function depending only on ${h_R}_j$. After performing factorization and rearranging the Eq. (6), we obtain

8 $$\begin{aligned} p_H^R\left( \mathbf{h}_\mathbf{R}\right) =\frac{1}{\eta ^{2K}}exp{\left( -\sum _{j=0}^{K-1}\frac{\psi ^2\left( {h_R}_j\right) }{2\eta ^2}\right) } \times U(min\ {h_R}_j)\prod _{j=0}^{K-1}{\psi ({h_R}_j)} \end{aligned}$$

where $U(h_R)$ is the unit step function. Now, by comparing the Eqs. (7) and (8), we can identify that $B\left( {h_R}_j\right) =\sum _{j=0}^{K-1}{\psi ^2\left( {h_R}_j\right) }$ is a sufficient statistics for estimation of velocity $(\upsilon )$. In next section, we make use of RBLS theorem to derive the MVU estimate of user velocity.

## Minimum variance unbiased velocity estimator

Prior to determining the MVU estimator, it is essential to establish the Rao-Blackwell-Lehmann-Scheffe (RBLS) theorem. This theorem states that if $\hat{\upsilon }$ serves as an unbiased estimator of $\upsilon$ and $B\left( {h_R}_j\right)$ represents an $(m \times 1)$ sufficient statistic for $\upsilon$, then $\hat{\upsilon }=E\left( \frac{\hat{\upsilon }}{B\left( {h_R}_j\right) }\right)$ qualifies as a valid unbiased estimator for $\upsilon$. Moreover, if the sufficient statistic is complete, then $\hat{\upsilon }$ is the MVU estimator.

First, we need to compute the expected value of the sufficient statistic $B\left( {h_R}_j\right)$, in order to determine the mean as,

9 $$\begin{aligned} E\left[ B\left( {h_R}_j\right) \right] =\sum _{j=0}^{K-1}{\psi ^2\left( {h_R}_j\right) }=K\left( \frac{\eta ^2\pi }{2}+\frac{4-\pi }{2}\eta ^2\right) . \end{aligned}$$

We could remove the bias of the component by dividing the statistic by *K*. It should be obvious that $B\left( {h_R}_j\right) =\sum _{j=0}^{K-1}{\psi ^2\left( {h_R}_j\right) }$ estimates the second moment and not the variance as desired. If we transform $B\left( {h_R}_j\right)$ as,

10 $$\begin{aligned} g\left( B\left( {h_R}_j\right) \right) =\frac{1}{K}\sum _{j=0}^{K-1}{\psi ^2\left( {h_R}_j\right) }-{\bar{\psi }}^2. \end{aligned}$$

Next, we take expectation of $g\left( B\left( {h_R}_j\right) \right)$ to find the mean value as,

11 $$\begin{aligned} E\left[ g\left( B\left( {h_R}_j\right) \right) \right] =E\left[ \frac{1}{K}\sum _{j=0}^{K-1}{\psi ^2\left( {h_R}_j\right) }-{\bar{\psi }}^2\right] =2\eta ^2-E\left[ {\bar{\psi }}^2\right] . \end{aligned}$$

After subsituting the value of $E\left[ {\bar{\psi }}^2\right] =\frac{1}{K}\left( \frac{\eta ^2\pi }{2}+\frac{4-\pi }{2}\eta ^2\right)$ in above equation we get,

12 $$\begin{aligned} E\left[ g\left( B\left( {h_R}_j\right) \right) \right] =2\eta ^2-\frac{1}{K}\left( \frac{\eta ^2\pi }{2}+\frac{4-\pi }{2}\eta ^2\right) =2\left( \frac{K-1}{K}\right) \eta ^2. \end{aligned}$$

If we multiply this statistics by $K/K-1$, it will then be unbiased for $\eta ^2$. Finally, the transformation is,

13 $$\begin{aligned} g\left( B\left( {h_R}_j\right) \right) =\frac{1}{K-1}\left[ \sum _{j=0}^{K-1}{\psi ^2\left( {h_R}_j\right) }-K{\bar{\psi }}^2\right] . \end{aligned}$$

However, since

14 $$\begin{aligned} & \sum _{j=0}^{K-1}{\left( \psi \left( {h_R}_j\right) -\bar{\psi }\right) ^2=}\sum _{j=0}^{K-1}{\psi ^2\left( {h_R}_j\right) }-2\sum _{j=0}^{K-1}\psi \left( {h_R}_j\right) \bar{\psi }\nonumber \\ & + K{\bar{\psi }}^2= \sum _{j=0}^{K-1}{\psi ^2\left( {h_R}_j\right) }-K{\bar{\psi }}^2 \end{aligned}$$

this may be written as,

15 $$\begin{aligned} g\left( B\left( {h_R}_j\right) \right) =\frac{1}{K-1}\left[ \sum _{j=0}^{K-1}\left( \psi ^2\left( {h_R}_j\right) -{\bar{\psi }}^2\right) ^2\right] . \end{aligned}$$

The reason the normalizing factor $1/(K-1)$ is utilized in the sample variance is due to the loss of one degree of freedom when estimating the mean, which results in a minimum variance unbiased (MVU) estimator. Our subsequent task, after establishing the RBLS theorem, is to identify a function *W* for which $\hat{\upsilon }=W(B)$ can serve as an unbiased estimator of $\upsilon$. Our process begins by calculating the expectation of the handover-count as,

16 $$\begin{aligned} E\left[ H^2\right] =\frac{1}{K}\sum _{j=0}^{K-1}{\psi ^2\left( {h_R}_j\right) }=0.41\upsilon T\sqrt{\lambda }+0.07+\left( \frac{4\upsilon T\sqrt{\lambda }}{\pi }\right) ^2. \end{aligned}$$

After additional simplification, we obtain,

17 $$\begin{aligned} \upsilon ^2+\frac{0.41\ \ \pi ^2}{16T\sqrt{\lambda }}\upsilon \ +\frac{\pi ^2}{16T^2\lambda }\left( 0.07-\frac{1}{K}\sum _{j=0}^{K-1}{\psi ^2\left( {h_R}_j\right) }\right) =0. \end{aligned}$$

Solving for the roots of $\upsilon$, we can express the MVU estimate of velocity $(\hat{\upsilon })$ as,

18 $$\begin{aligned} \hat{\upsilon }=-\frac{0.41\ \ \pi ^2}{32T\sqrt{\lambda }}+\frac{\pi }{4T\sqrt{\lambda }}\times \sqrt{\frac{1}{K}\sum _{j=0}^{K-1}{\psi ^2\left( {h_R}_j\right) }-0.07-\frac{{0.41}^2\ \ \pi ^2}{64}}. \end{aligned}$$

The MVU estimate of user velocity, as represented in Eq. (18), is a non-linear function of handover count samples. Therefore, we utilized the statistical linearization argument described in^36^ to assess the bias of the estimator. Additionally, let *Y* be a function such that,

19 $$\begin{aligned} \hat{\upsilon }=Y(\delta ) \end{aligned}$$

where, $\delta \ =\frac{1}{K}\sum _{j=0}^{K-1}{\psi ^2\left( {h_R}_j\right) }$. Thus, function $Y(\delta ))$ can be defined as

20 $$\begin{aligned} Y(\delta )=-\frac{0.41\ \ \pi ^2}{32T\sqrt{\lambda }}+\frac{\pi }{4T\sqrt{\lambda }}\sqrt{\delta -0.07+\frac{0.41\ \ \pi ^2}{16}}. \end{aligned}$$

**Fig. 4:**
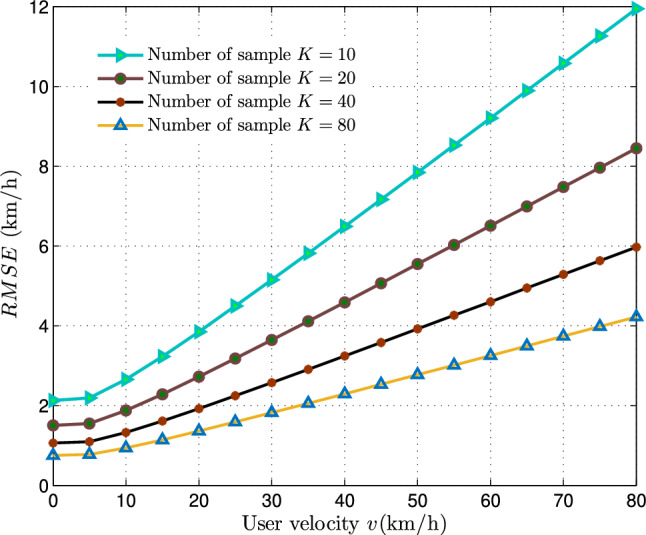
RMSE for MVU estimator across user velocity $(\upsilon )$ (*km*/*h*), $T = 8s$, and $\lambda =100\ { BSs/km}^2$.

Utilizing the independent and identically distributed (i.i.d.) characterization of the handover-count and linearizing around $\delta _0\ =E\left[ \frac{1}{K}\sum _{j=0}^{K-1}{\psi ^2\left( {h_R}_j\right) }\right] =\ \frac{\eta ^2\pi }{2}+\frac{4-\pi }{2}\eta ^2$, yields the following result,

21 $$\begin{aligned} Y(\delta )\approx \ Y(\delta _0)+\left( \frac{dY(\delta )}{d\delta }\right) _{\delta =\delta _0}\left( \delta -\delta _0\right) \end{aligned}$$

where, $\frac{dY(\delta )}{d\delta }=\frac{\pi }{8T\sqrt{\lambda }}\frac{1}{\sqrt{\delta -0.07+\frac{{0.41}^2\ \ \pi ^2}{64}}}$, after substitution and simplifying further, we get

22 $$\begin{aligned} \hat{\upsilon }\approx \upsilon +\frac{\frac{\pi }{8T\sqrt{\lambda }}}{\left( \frac{4\upsilon T\sqrt{\lambda }}{\pi }+\frac{0.41\pi }{8}\right) }\left( \frac{1}{K}\sum _{j=0}^{K-1}{\psi ^2\left( {h_R}_j\right) }-2\eta ^2\right) . \end{aligned}$$

As *K* increases towards infinity i.e $K\rightarrow \infty$, the estimated velocity $\hat{\upsilon }$ asymptotically follows the mean:

23 $$\begin{aligned} E\left[ \ \hat{\upsilon }\right] \approx \upsilon . \end{aligned}$$

Therefore, we can conclude that the MVU velocity estimator proposed, based on the handover count PMF approximated via Rayleigh distribution, is asymptotically unbiased. Subsequently, the variance of the proposed estimator can be expressed as,

24 $$\begin{aligned} var\left( \hat{\upsilon }\right) =\left[ \frac{\pi }{8T\sqrt{\lambda }}\frac{1}{\left( \frac{4\upsilon T\sqrt{\lambda }}{\pi }+\frac{0.41\ \ \pi }{8}\right) }\right] ^2\ \frac{1}{K}\ var(\psi ^2\left( h_R\right) ) \end{aligned}$$

where, $\ var(\psi ^2\left( h_R\right) )$ is derived in Appendix A and the variance of the proposed estimator can be expressed as,

25 $$\begin{aligned} var\left( \hat{v}\right) =\frac{1}{4K}{\ \left( 8-\frac{\pi ^2}{2}\right) \left[ \frac{\pi }{8T\sqrt{\lambda }}\frac{1}{\left( \frac{4\upsilon T\sqrt{\lambda }}{\pi }+\frac{0.41\ \ \pi }{8}\right) }\right] }^2 \left( 0.41\upsilon T\sqrt{\lambda }+\left( \frac{4\upsilon T\sqrt{\lambda }}{\pi }\right) ^2+0.07\right) ^2. \end{aligned}$$

**Fig. 5:**
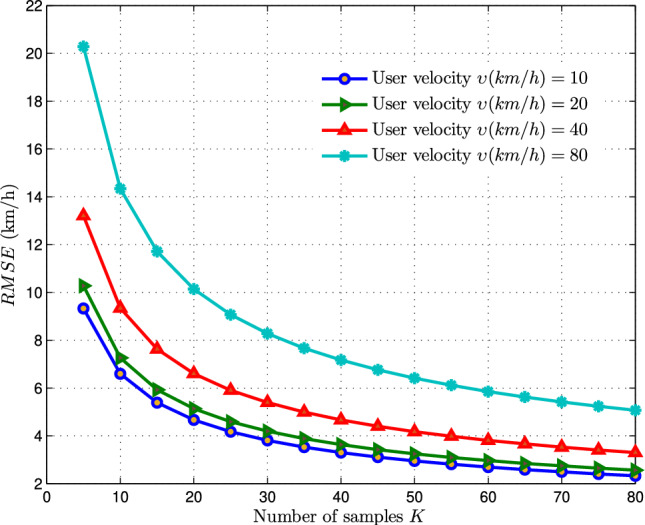
RMSE of MVU estimator in relation to number of both vertical and horizontal handover count samples (*K*).

**Fig. 6:**
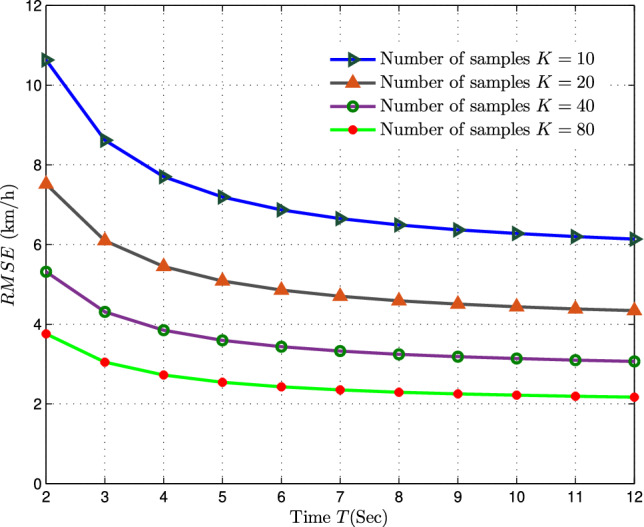
Assessing RMSE Variations of MVU Estimator with Time Span (*T*) at $\lambda = 100BSs/km^2$.

## Cramer-Rao lower bound for quantifying estimation accuracy

Unbiased velocity estimators are defined as those which, on average, yield the true value of the user velocity. The CRLB establishes the minimum achievable variance for an unbiased estimator, indicating that any unbiased estimator with a variance lower than the CRLB is physically unattainable. Therefore, it serves as a benchmark with which we can evaluate the performance of any unbiased estimator. An unbiased estimator whose variance reaches the CRLB is referred to as an efficient estimator.

Given the Rayleigh-approximated joint PMF of *K* handover count samples presented in Eq. (6), we can derive the CRLB for the velocity estimator based on the handover-count PMF approximated via the Rayleigh distribution as,

26 $$\begin{aligned} CRLB\ge \frac{1}{I(v)} \end{aligned}$$

where *I*(*v*) is the fisher information and can be computed as,

27 $$\begin{aligned} I(v)=-E\left( \frac{\partial ^2\ }{\ \partial \upsilon ^2}ln\ p_H^R\left( \mathbf{h}_R\right) \right) \end{aligned}$$

The Fisher Information Matrix *I*(*v*) is derived in Appendix B and CRLB can be expressed as,

28 $$\begin{aligned} CRLB=\frac{\eta ^4}{K\left( \frac{\partial \eta ^2}{\partial \upsilon }\right) ^2} \end{aligned}$$

Differentiating Eq. (3) with respect to *v* yields $\frac{\partial \eta ^2}{\partial \upsilon }=\frac{1}{2}\left( 0.41T\sqrt{\lambda }+\frac{32T^2\lambda \upsilon }{\pi ^2}\right)$, and substituting the value of $\eta ^2$ from Eq. (3) in Eq. (28) we get,

29 $$\begin{aligned} CRLB=\frac{\left( \frac{1}{2}\left( 0.41\upsilon T\sqrt{\lambda }+\left( \frac{4\upsilon T\sqrt{\lambda }}{\pi }\right) ^2+0.07\right) \right) ^2}{K\left( \frac{1}{2}\left( 0.41T\sqrt{\lambda }+\frac{32T^2\lambda \upsilon }{\pi ^2}\right) \right) ^2} \end{aligned}$$

## Result analysis

We utilize the root mean square error (RMSE) as an evaluation metric for the proposed MVU estimator’s performance, derived from both vertical and horizontal handover counts approximated via Rayleigh distribution. The equation $RMSE=\sqrt{variance}$ is employed to showcase the validity of our analysis and to facilitate a comparison with the respective CRLB in assessing the performance of the proposed MVU estimator.

The variance of the proposed MVU estimator, which is based on the PMF of vertical and horizontal handover-counts approximated using the Rayleigh distribution, is represented by Eq. (25). In Fig. 4, the RMSE plot of the proposed MVU estimator demonstrates the impact of increasing user velocity (*v*) for different numbers of samples (*K*) (10, 20, 40, 80) over a fixed measurement time-span ($T = 8s$) and base station density ($\lambda = 100BSs/km^2)$. The plot illustrates that as the user velocity $\upsilon$ increases, the RMSE of the proposed MVU estimator also increases, suggesting that the estimator delivers optimal results when the user moves slowly, while its performance diminishes as the user’s speed increases.

**Fig. 7:**
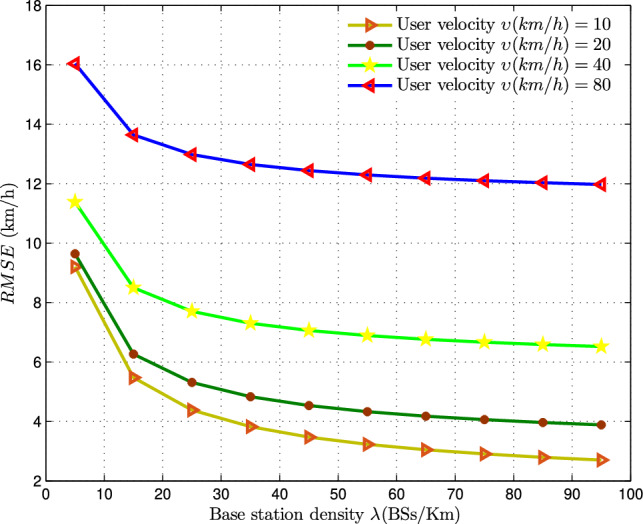
Analyzing the effect of base station density $(BSs/km^2)$ on RMSE of MVU estimator at $T = 8s$.

Figure 5 illustrates the fluctuation of RMSE for the proposed MVU estimator at a constant base station density of $\lambda =10\,\text{BSs/km}^2$ and a measurement time of $T=8s$ across various velocities ($\upsilon =10,20,40,80\ { km/h}$). It is also evident that the RMSE diminishes as the number of handover-count samples increases, indicating that greater sampling leads to additional information for estimation and subsequently lowers the error.

The graph depicted in Fig. 6 displays the RMSE variation for the proposed MVU estimator across a time span (*T*), maintaining a fixed BS density of $\lambda =100\,{ BSs/km}^2$ and a velocity of $v=40\,{ km/h}$. It is apparent that the RMSE of the MVU estimator diminishes as the time span for measurement increases. For example, when the service provider utilizes $K=20$ samples and $\lambda =100\,{ BSs/km}^2$, the RMSE for $T=3s$ and $T=12s$ are 6*km*/*h* and 4.2*km*/*h* respectively, showcasing an improvement in accuracy with a longer time span. However, it’s important to note that a longer time span leads to enhanced accuracy but also results in a slower response from the estimator, indicating a trade-off between accuracy and the duration allocated for data collection.

By employing a fixed time span of $T = 8s$ for measuring vertical and horizontal handover counts, along with $K = 10$ samples, we can create a plot displaying RMSE against BS density $\lambda$ in Fig. 7. This analysis encompasses various user velocities $(\upsilon =10, 20, 40, 80\,{ km/h})$. In a specific instance where the user’s velocity is $\upsilon = 20\,{ km/h}$ and a fixed time span of $T = 8s$ is utilized for handover-count measurement, the RMSE of the MVU-based velocity estimator is observed to be 8*km*/*h* and 6.4*km*/*h* for BS densities of $\lambda =20\, BSs/km^2$ and $\lambda =90\,{ BSs/km}^2$, respectively. From this, we can deduce that an increase in BS density $(\lambda )$ leads to a reduction in the variance of the velocity estimator, thereby enhancing the accuracy of velocity estimation in hyper-dense networks.

**Fig. 8:**
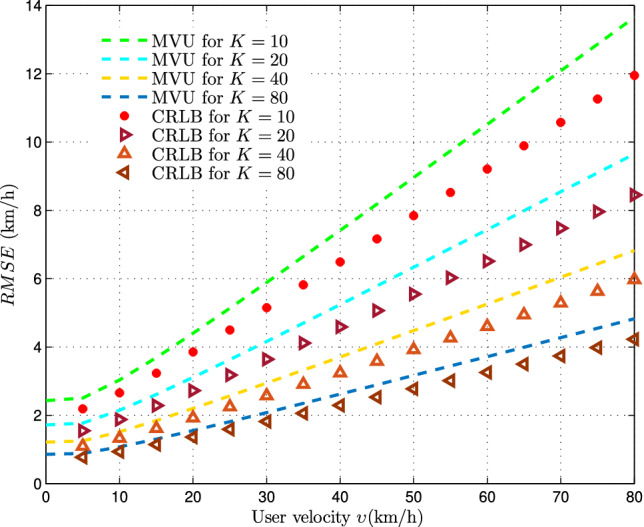
RMSE of the MVU estimator compared to the CRLB with increasing user velocity (*v*), at fixed $\lambda =10\,0BSs/km^2$, and $T = 8s$.

Subsequently, we conduct an analysis on the precision of MVU estimates derived from vertical and horizontal handover-count statistics. This is achieved through a comparison of the proposed estimator variance with its corresponding Cramér-Rao Lower Bound (CRLB), which represents the minimum variance achievable for an unbiased estimator and is formulated in Eq. (29). Figure 8 illustrates the relationship between the CRLB and the proposed estimator variance as user velocity $(\upsilon )$ increases, considering different sample sizes for estimation $(K=10,20,40,80)$. We have held the base-station density and handover-count measurement time-span at $\lambda =100BSs/km^2$ and $T=8s$ respectively. It is evident that as the mobile user velocity rises, the CRLB similarly increases. Furthermore, the RMSE of the MVU estimator consistently aligns closely with the CRLB as the number of handover count samples utilized for estimation increases.

### Adaptation of MVU velocity estimation under co-channel conditions

To reflect real-world conditions in HetNets, we have incorporated the scenario of co-channel deployment, where both macro-cell BSs and small-cell BSs operate on the same frequency band. This approach, which is increasingly used to maximize area spectral efficiency, results in cell boundaries that are dynamic and sometimes indistinct, influenced by factors such as BS density, variations in transmit power, and load-balancing bias settings. These complexities contribute to elevated levels of co-channel interference, particularly near cell edges, which can cause unnecessary or ping-pong handovers and add challenges to mobility management. To maintain the effectiveness of the MVU velocity estimation method under these conditions, our framework has been enhanced to model handover counts using stochastic geometry, taking into account random base station placement, user velocity, network density, transmit power, and observation duration. Our results demonstrate that the MVU estimator remains reliable when BS coverage boundaries are clearly delineated, although its variance may increase in environments with high interference or rapidly changing network conditions.

## Conclusion

In this paper, we have introduced a Minimum Variance Unbiased velocity estimator for user velocity in HetNets, incorporating both horizontal and vertical handover counts. We have modeled the PMF of handover counts as Rayleigh distributed, showing minimal MSE with simulated PMF. Subsequently, we extracted sufficient statistics for velocity estimation using the Neyman-Fisher factorization theorem and derived the MVU estimator through the RBLS theorem. Our study included the derivation of the CRLB for velocity estimation and compared it to the variance of the proposed MVU estimator, demonstrating a close alignment between the MVU estimator’s variance and the CRLB. Furthermore, we observed that the variance of the MVU estimator decreases with an increase in the number of handover-count samples, indicating that a higher quantity of samples yields more information for estimation. Additionally, we found that the variance of the MVU velocity estimator decreases as the time period used for handover-count measurement increases, suggesting a trade-off between accuracy and speed in velocity measurement.

## Data Availability

No pre-existing datasets were used in the performance of this study. The datasets generated during the current study are available from the corresponding author upon reasonable request.

## Appendix A: Variance of $\psi ^2$

Here, we derive the expression for the variance of $\psi ^2\left( h_R\right)$, denoted as $var(\psi ^2\left( h_R\right) )$, and it can be expressed as,

30 $$\begin{aligned} \ var(\psi ^2\left( h_R\right) )=4\times {Mean\left( h_R\right) }^2\times var\left( h_R\right) +2\times {var\left( h_R\right) }^2. \end{aligned}$$

where, $\ var\left( h_R\right) =\frac{\left( 4-\pi \right) }{2}\eta ^2$ and $Mean\left( h_R\right) =\ \eta \sqrt{\frac{\pi }{2}}$, substituting these values in Eq. (30) we get,

31 $$\begin{aligned} \ var(\psi ^2\left( h_R\right) )=4\eta ^2\frac{\pi }{2}\times \frac{\left( 4-\pi \right) }{2}\eta ^2+2\left( \frac{4-\pi }{2}\right) ^2\eta ^4. \end{aligned}$$

After further simplification of the equation, we obtain,

32 $$\begin{aligned} \ var(\psi ^2\left( h_R\right) )=\left( 8-\frac{\pi ^2}{2}\right) \times \eta ^4. \end{aligned}$$

Upon substituting the value of $\eta$ from Eq. (3) into Eq. (32), we obtain,

33 $$\begin{aligned} \ var(\psi ^2\left( h_R\right) )=\frac{1}{4}\left( 8-\frac{\pi ^2}{2}\right) \times \left( \left( \frac{4\upsilon T\sqrt{\lambda }}{\pi }\right) ^2+0.41\upsilon T\sqrt{\lambda }+0.07\right) ^2. \end{aligned}$$

## Appendix B: Fisher Information Matrix *I*(*v*)

Consider the joint PMF of *K* handover count samples as expressed in Eq. (6) and taking logarithm on both sides we get the log-likelihood function as,

34 $$\begin{aligned} {ln\ p}_H^R\left( \mathbf{h}_R\right) =K\ ln\left( \frac{\psi ({h_R}_j)}{\eta ^2}\right) -\sum _{j=0}^{K-1}\frac{\psi ^2\left( {h_R}_j\right) }{2\eta ^2} \end{aligned}$$

Now, differentiate the above log-likelihood function with respect to $\upsilon$, to get first derivative as,

35 $$\begin{aligned} \frac{\partial }{\partial \upsilon }ln\ p_H^R\left( \mathbf{h}_R\right) =-\frac{K}{\eta ^2}\frac{\partial \eta ^2}{\partial \upsilon }+\frac{1}{2\eta ^4}\frac{\partial \eta ^2}{\partial \upsilon }\sum _{j=0}^{K-1}{\psi ^2\left( {h_R}_j\right) } \end{aligned}$$

Next, differentiate the Eq. (35) with respect to $\upsilon$, to get second derivative as,

36 $$\begin{aligned} & \frac{\partial ^2\ }{\ \partial \upsilon ^2}ln\ p_H^R\left( \mathbf{h}_R\right) =-\frac{K}{\ \eta ^2}\frac{\partial ^2\eta ^2}{\partial \upsilon ^2}+\frac{K}{\ \eta ^4}\left( \frac{\partial \eta ^2}{\partial \upsilon }\right) ^2 \nonumber \\ & -\frac{1}{\eta ^6}\left( \frac{\partial \eta ^2}{\partial \upsilon }\right) ^2\sum _{j=0}^{K-1}{\psi ^2\left( {h_R}_j\right) }+\frac{1}{{2\eta }^4}\frac{\partial ^2\eta ^2}{\partial \upsilon ^2}\sum _{j=0}^{K-1}{\psi ^2\left( {h_R}_j\right) } \end{aligned}$$

The Fisher Information matrix *I*(*v*) can thus be expressed as,

37 $$\begin{aligned} & I(v)=-\ E\left( \frac{\partial ^2\ }{\ \partial \upsilon ^2}ln\ p_H^R\left( \mathbf{h}_R\right) \right) =\frac{1}{\ \eta ^2}\left( \frac{\partial ^2\eta ^2}{\partial \upsilon ^2}\left( K-\frac{1}{{2\eta }^2}\sum _{j=0}^{K-1}E\left[ \psi ^2\left( {h_R}_j\right) \right] \right) \right) \nonumber \\ & -\frac{1}{\ \eta ^4}\left( \frac{\partial \eta ^2}{\partial \upsilon }\right) ^2\left( K-\frac{1}{\eta ^2}\sum _{j=0}^{K-1}E\left[ \psi ^2\left( {h_R}_j\right) \right] \right) \end{aligned}$$

As $E\left[ \psi ^2\left( {h_R}_j\right) \right] ={2\eta }^2$, after substitution and further simplification we can rewrite fisher information matrix as,

38 $$\begin{aligned} I(v)=\ \frac{K}{\ \eta ^4}\left( \frac{\partial \eta ^2}{\partial \upsilon }\right) ^2 \end{aligned}$$

## Code availability

Yes.
